# Oral shea nut oil triterpene concentrate supplement ameliorates pain and histological assessment of articular cartilage deterioration in an ACLT injured rat knee osteoarthritis model

**DOI:** 10.1371/journal.pone.0215812

**Published:** 2019-04-19

**Authors:** Ing-Jung Chen, Sheng-Hsiung Lin, Chih-Shung Wong

**Affiliations:** 1 Department of Medical Research, Cathay General Hospital, Taipei, Taiwan; 2 Department of Anesthesiology, Cathay General Hospital, Taipei, Taiwan; 3 Planing and Management Office, Tri-Service General Hospital, Taipei, Taiwan; 4 Graduate Institute of Medical Sciences, National Defense Medical Center, Taipei, Taiwan; Ain Shams University, EGYPT

## Abstract

Osteoarthritis (OA) is a multifactorial joint disease and a common disabling condition in the elderly population. The associated pain and pathohistological changes in cartilage are common features of OA in both humans and animal models. Shea nut oil extract (SheaFlex75) contains a high triterpenoid concentration and has demonstrated anti-inflammatory and antiarthritic effects in both human and animal studies. In this study, we aim to investigate the potential of SheaFlex75 to prevent articular cartilage deterioration in a rat model of chronic OA progression. By employing anterior cruciate ligament transection (ACLT) with medial meniscectomy (MMx)-induced OA, we found attenuation of both early and chronic onset OA pain and cartilage degeneration in ACLT+MMx rats receiving SheaFlex75 dietary supplementation. Under long-term oral administration, the rats with induced OA presented sustained protection of both pain and OA cartilage integrity compared to the OA-control rats. Moreover, rats subjected to long-term SheaFlex75 ingestion showed normal biochemical profiles (AST, BUN and total cholesterol) and presented relatively lower triglycerides (TGs) and body weights than the OA-control rats, which suggested the safety of prolonged use of this oil extract. Based on the present evidence, preventive management is advised to delay/prevent onset and progression in OA patients. Therefore, we suggest that SheaFlex75 may be an effective management strategy for symptom relief and cartilage protection in patients with both acute and chronic OA.

## Introduction

Osteoarthritis (OA) is a multifactorial joint disease and a common disabling condition of the global population [1, 2]. Although disease progression is slow, the associated joint pain and stiffness lead to reduced physical function and quality of life and frequent physician visits by the affected population [3, 4]. The knee joint is the most affected site of OA in aged people, and the incidence increases with age [5]. According to a case-control study report, 69% of first knee OA consultations occur between 55 and 74 years of age, and the prevalence of knee OA is estimated to be 12.5% in those aged ≥45 years [6]. At least one episode of knee pain is reported in approximately 25% of individuals aged over 55 years [7] each year. In addition to aging, other major risk factors associated with knee OA are obesity and previous knee trauma [8]. Management of the associated factors is implemented as an important OA treatment strategy.

The main OA treatment goals are reduction of pain, improvement of physical function and quality of life [9, 10], which are also the primary outcome assessments of clinical studies for drug development for OA [11, 12]. Additionally, OA treatment seeks to modify the underlying joint structure. However, measurements of OA structural changes using conventional radiographs are not well-defined, and the severity of radiographic changes may not be directly associated with patients’ clinical presentations [13–15]. The laboratory animal model of OA has been widely used to support the assessment of this therapeutic modification and the pathohistological changes of OA [16]. An interventional animal study allows researchers to examine tissue specimens and provides quantitative measurement of cartilage damage and the underlying mechanism of the target of interest.

Shea butter contains a high triterpene concentration and is considered to have anti-inflammatory and antioxidant properties [17]. Previous reports demonstrated both in vivo and in vitro anti-inflammatory effects of triterpenes (e.g., lupeol and β-amyrin) [18–20]. SheaFlex75 is a patented nutraceutical product with a specific formulation of triterpene concentrates that is prepared from the African shea tree *Vitellaria paradoxa*. This natural supplement was shown to reduce OA pain in clinical studies investigating long-term usage [21, 22]. In addition, our previous report showed that preventive oral administration of SheaFlex75 reduces cartilage degeneration in anterior cruciate ligament transection plus medial meniscectomy (ACLT+MMx) surgery-induced OA rats [23].

In this study, we examined the potential effect of SheaFlex75 on attenuation of disease progression of both early and chronic osteoarthritis rats and elucidated the safety of this natural product under long-term use.

## Materials and methods

### Osteoarthritis animal model

All animal care and experimental protocols complied with institutional and international standards (Principles of Laboratory Animal Care, National Institutes of Health) and were approved (IACUC-17-093) by the Institutional Animal Care and Use Committee of the National Defense Medical Center (Taipei, Taiwan). Adult male Wistar rats were purchased from BioLASCO Taiwan Co., Ltd. (Yilan, Taiwan) and kept in animal cages with free access to the standard diet and water. The room had a 12-h light/dark cycle and a temperature of 22±2°C with 55% humidity.

The ACLT+MMx was performed to induce OA in the right knee of the rats. Briefly, male Wistar rats (330–350 g) were anesthetized in an induction chamber using 5% isoflurane and then maintained with 2% isoflurane via a custom-made facemask. The right knee joint skin was shaved, and the area was sterilized with 70% isopropyl alcohol and iodine solution. An incision was made in the medial aspect of the joint capsule (anterior to the medial collateral ligament); the anterior cruciate ligament was transected, and the medial meniscus was removed completely. Following surgery, the joint was irrigated with normal saline, and the joint capsule was sutured with 4–0 vicryl and 4–0 nylon for skin closure. Next, an iodine solution was used to sterilize the wound area, and cefazolin (100 mg/kg/day) was administered intramuscularly for 3 days to prevent infection. After recovery from anesthesia, all rats were returned to their cages. For the sham-operated rats, an incision was made at the medial aspect of the joint capsule to expose the anterior cruciate ligament, but neither ACLT nor removal of the medial meniscus was performed.

### Experimental design and oral SheaFlex75 administration

**Fig 1:**
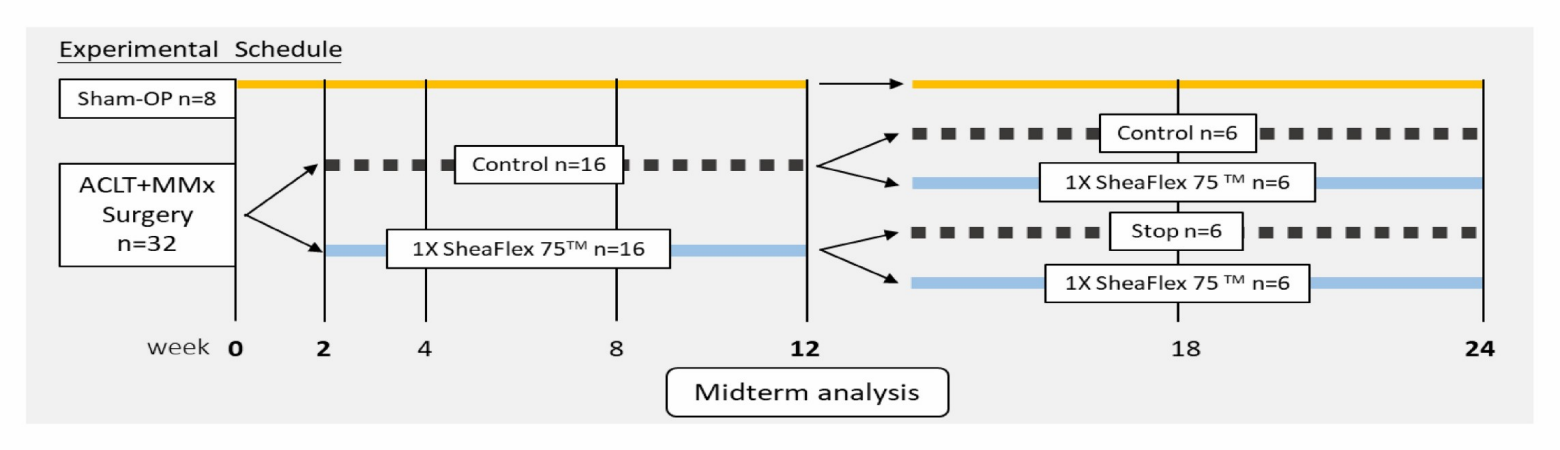
Experimental design and the time course of the study.

As shown in Fig 1, the ACLT+MMx (n = 32), or sham surgery (n = 8), was performed at week 0. The body weights, widths of the knee joints and weight-bearing test results were measured before the surgery as the baseline. Fourteen days after surgery, the animals were randomly assigned to the non-feed control (OA-control, n = 16) or the SheaFlex75 1X (OA-SNO 1X, n = 16) (223.2 mg/kg) feed group for 10 weeks. The SheaFlex75 oil extract (Universal Integrated Corp., Taipei, Taiwan) was administered by oral gavage with the aid of isoflurane anesthesia.

Midterm knee joint samples were taken from sham-OP (n = 2), OA-control (n = 4) and OA-SNO 1X (n = 4) rats at week 10 after treatment initiation. Then, the OA-control rats were reassigned to SheaFlex75 oral administration (OA-Late SNO 1X, n = 6) or continued as OA controls (n = 6) for another 12 weeks. In contrast, treatment in the OA-SNO 1X treated rats was either discontinued (OA-SNO-stop, n = 6) or continued with SheaFlex75 1X (OA-SNO 1X, n = 6) for 12 weeks. After the tests were completed at the end-point of the study (week 24 post-surgery), the rats were sacrificed, and their right knees were dissected for histologic assessment.

### Knee width and weight-bearing test

The width of the knee joint was measured using a steel caliper (resolution 0.01 mm, SV-03-050, E-Base Measuring Tools Co., Taiwan) every week after the operation, and the width of the contralateral knee was used as the naïve control. The data are expressed as the Δ knee width (mm); the value is derived from the OA rats (knee width difference of the operated knee and naïve knee) minus the mean value of the sham-OP rats (knee width difference of the operated knee and naïve knee) and calculated as the actual joint swelling induced by ACLT+MMx.

Hind paw static weight-bearing was measured using an incapacitance tester (Linton Instrumentation, Norfolk, UK) to detect OA-induced changes in postural equilibrium every two weeks. The rats were placed on their hind paws in a box containing an inclined plane (65° from horizontal) that was placed above the incapacitance apparatus. After a brief accommodation period, the weight that the animals applied to each hind limb was measured independently by the apparatus. Five measurements were taken and averaged for each rat. The data are expressed as the difference between the weight applied to the naïve hind limb and the weight applied to the operated hind limb (Δ weight, g); the change in the weight distribution is associated with the OA pain in the rats [24, 25].

### Histopathological examination of the joints

After the tests were completed, the rats were sacrificed via exsanguination under deep anesthesia. The OA knee joints were removed and fixed in 10% formalin for 2 days, followed by decalcifier solution based on EDTA disodium (12.5%, pH 7.0) for ~4 weeks. After decalcification, the joints were embedded in paraffin blocks, and histological coronal sections were obtained. Hematoxylin and eosin (H&E) and Toluidine blue/fast green staining were used to examine morphological changes. The severity of articular cartilage damage was evaluated using the modified Osteoarthritis Research Society International (OARSI) scoring system [26]. The cartilage matrix loss width (0%, 50%, and 100%), cartilage degeneration score, total and significant cartilage degeneration widths, and zonal depth ratio of the lesions were evaluated.

### Liver, kidney and lipid profiles

Blood samples were taken from the rat tail vein at week 4, 8 and 12 post-ACLT+MMx surgery. The blood was centrifuged (8000 x g for 5 min) to separate sera and stored in a -80°C freezer prior to analysis. The serum AST, BUN, total cholesterol (T-CHO), high-density lipoprotein (HDL) and TG levels were measured using the FUJI DRI-CHEM 4000i (FUJIFILM Corporation, Tokyo, Japan) at the Taiwan Mouse Clinic (Academia Sinica, Taipei, Taiwan).

### Statistical analysis

The data are expressed as the mean±S.E.M. Graphical representations and statistical calculations were aided by GraphPad Prism version 6.01. Shapiro–Wilk test was used to check the normal distribution of data. Two-way ANOVA, Tukey’s multiple comparisons test, Sidak's multiple comparisons test and Student's t-test were used to analyze the data.

## Results

### Oral SheaFlex75 administration reduces OA-induced knee joint swelling and weight bearing asymmetry

**Fig 2:**
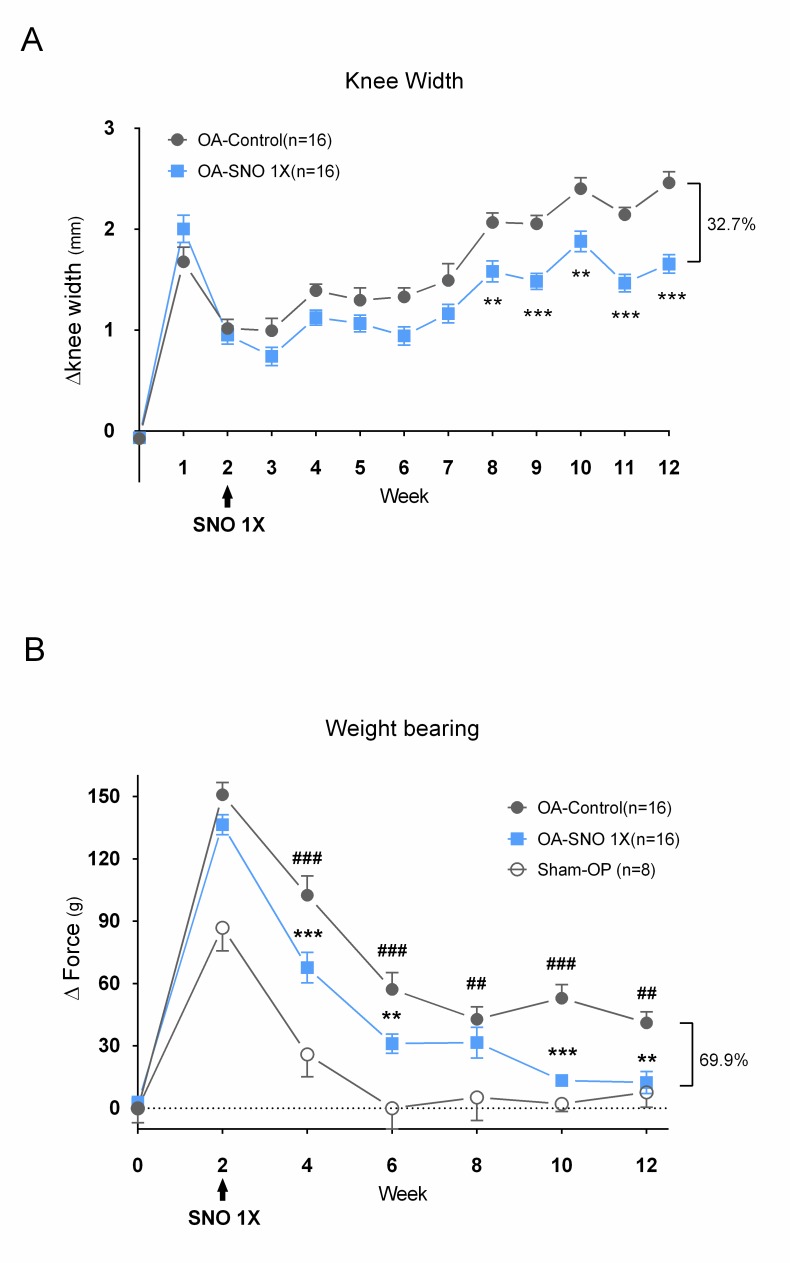
Oral SheaFlex75 administration reduces acute OA-induced knee joint swelling and restores the hind paw weight-bearing imbalance. ACLT+MMx was performed to induce knee OA. Two weeks later, the rats were divided into the OA-control and SheaFlex75 (OA-SNO 1X) (223.2 mg/kg) treatment groups for 10 weeks. (A) Weekly knee width measurements of the OA-control and OA-SNO 1X rats. The data are presented as the Δ knee width (mm) and expressed as the mean±S.E.M. and two-way ANOVA and Sidaks’s multiple comparisons test were used to analyze the data. Asterisks denote significant differences between the OA-SNO 1X and OA-control groups: *P<0.05, **P<0.01, ***P<0.001. (B) Weight-bearing test of the sham-OP, OA-control and OA-SNO 1X rats. The data are presented as the Δ force (g) of both hind paws, and the values are expressed as the mean±S.E.M. and two-way ANOVA and Tukey’s multiple comparisons test were used to analyze the data. Number signs denote significant differences between the OA-control and sham-OP groups, and asterisks denote significant differences between the OA-SNO 1X and OA-control groups: # or *P<0.05, ## or **P<0.01, ### or ***P<0.001.

Two weeks after ACLT+MMx surgery, the rats were randomly assigned to the OA-control and OA-SNO 1X (223.2 mg/kg) groups for 10 weeks. In the first week following the operation, we found a peak of joint swelling due to acute inflammation induced by the surgery. Subsequently, in the OA-control group, we found that ACLT+MMx induced a constant and gradually increasing knee width that reflected progressive knee joint inflammation and accumulation of synovial fluid. In contrast, long-term oral administration of SheaFlex75 reduced the OA-induced knee joint swelling as early as 4 weeks post-treatment and achieved a difference of approximately 32.7% (Δ width: 1.656±0.091 vs 2.460±0.111 mm) at week 10 (Fig 2A). The OA-induced pain was assessed using the weight-bearing test to evaluate postural equilibrium under unilateral knee joint pain. Early inflammation was induced by the surgery with a peak value obtained at the first assessment. Then, we found sustained instability from 6 to 12 weeks in the OA-control groups, whereas the sham-OP rats quickly recovered to their baseline (Fig 2B). The significant difference between the OA-control and OA-SNO 1X groups was observed as early as two weeks into the regimen. After 10 weeks of administration, the hind paw weight bearing of the OA-SNO 1X group rats showed a 69.9% reduction compared to the OA-control group rats (Δ force: 12.342±5.352 g vs 41.066±5.320 g) and reached a balance similar to that of the sham-OP group rats (Δ force = 7.579±7.111 g).

### SheaFlex75 sustainably reduces pain and cartilage deterioration in chronic OA rats

**Fig 3:**
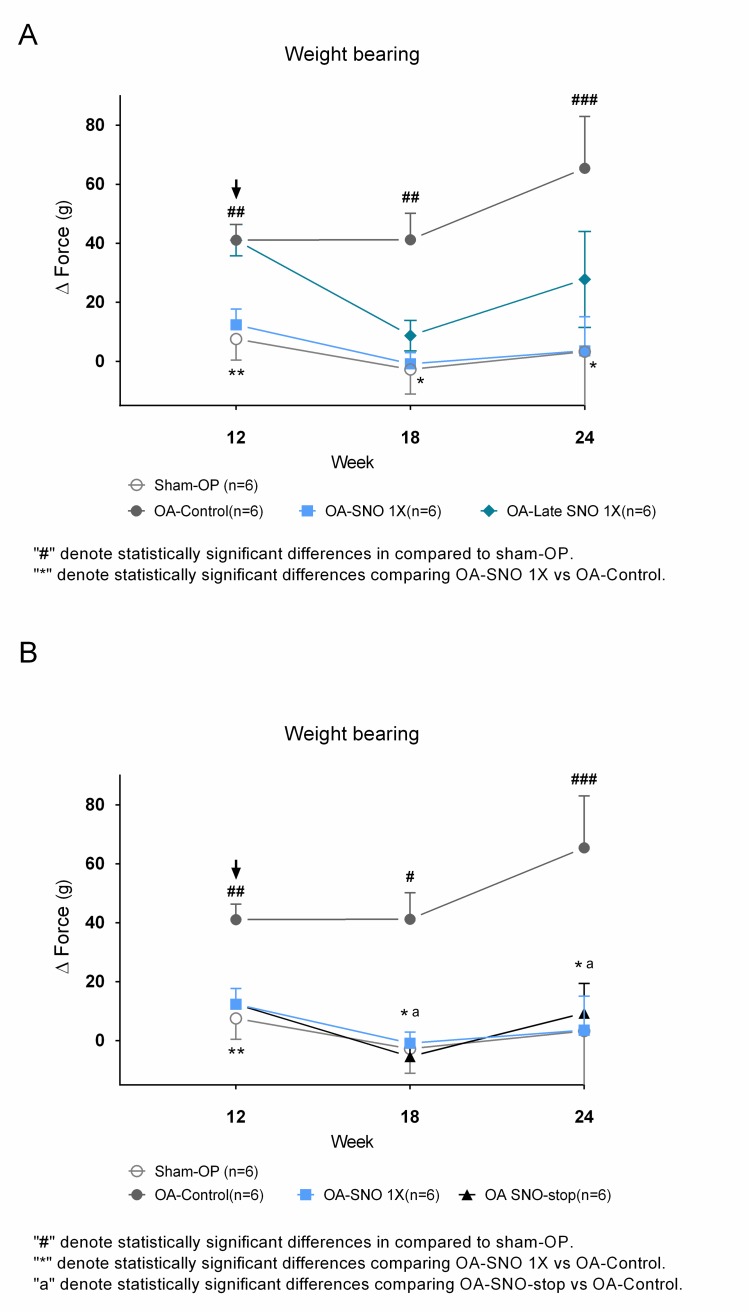
Long-term use of SheaFlex75 sustainably reduces chronic OA symptomatology. (A) Weight-bearing test results after long-term use of SheaFlex75 (OA-Late SNO 1X, 12 weeks post-surgery), OA-SNO 1X and OA-control group compared to the sham-OP group. (B) Weight-bearing test results for the discontinued treatment (OA-SNO-stop), OA-SNO 1X and OA-control group compared to the sham-OP group. Arrows indicate the switch point of treatment. Data are presented as the Δ force (g) of both hind paws; the values are expressed as the mean±S.E.M. and two-way ANOVA and Tukey’s multiple comparisons test were used to analyze the data.

To evaluate the efficacy of oral SheaFlex75 ingestion in chronic OA rats, the OA-control group was further divided into two groups; the first group continued as the OA-control group, and the second group started oral administration of SheaFlex75 (OA-Late SNO 1X) for 12 weeks. Fig 3A shows that the weight bearing of both groups was significantly different from that of the sham-OP and OA-1X SNO groups at week 12 post-ACLT+MMx. Unsurprisingly, the OA-Late SNO 1X group showed relief of the weight-bearing imbalance after 6–12 weeks of SNO administration (i.e., weeks 18–24 post-surgery), whereas the OA-control rats still presented a high level of imbalance at weeks 18–24 post-surgery; the OA-SNO 1X group achieved better weight-bearing test results during the entire experimental course (Fig 3A). Additionally, the previously OA-SNO 1X-treated rats were divided into 2 groups; the first group continued with the same 1X dose, and the second group discontinued the daily administration (OA-SNO-stop). Fig 3B shows a significant reduction in weight-bearing imbalance in the 2 groups compared to the OA-control group. Unexpectedly, we still found relatively balanced weight bearing in the OA-SNO-stop group, which suggested a prolonged protective effect on structural integrity after a long-term full regimen of the shea nut oil supplement. However, the subsequent increasing trend that was observed in the OA-SNO-stop group at week 24 indicated a disadvantage of discontinuation of the supplement against administration of the full regimen, as shown in Fig 3B.

Tibia cartilage damage was assessed at weeks 12 and 24 post-surgery. As shown in Table 1, the ACLT+MMx-operated (OA-control) rats presented a significant increase in the analyzed parameters with increasing severity from week 12 to 24, demonstrating progressive OA damage over time (Table 1). Unsurprisingly, the SheaFlex75-treated OA rats exhibited less matrix loss (0% width, 1.953±0.132 mm vs 1.615±0.138 mm, p = 0.0495) and a significant cartilage degeneration width (1.123±0.123 mm vs 0.734±0.184 mm, p = 0.0442) at the end of the study, suggesting that less severe cartilage matrix loss and degeneration occurred following ACLT+MMx-induced OA. Moreover, other OARSI scores (e.g., cartilage matrix loss (100% width, p = 0.0745) and total cartilage degeneration width (p = 0.0747)) were marginally reduced in the OA-SNO 1X rats. Consistent with the weight-bearing results, we found a significant reduction in the total/significant cartilage degeneration width in the OA-Late SNO groups in compared to OA-control, which demonstrated that protection against chronic OA progression was achieved after long-term use of SheaFlex75. In accordance with the weight-bearing data, discontinuation of SheaFlex75 (after 10 weeks of the full regimen) still showed protection on the total cartilage degeneration score compared to the OA-control rats. However, the lack of a significant reduction in the significant cartilage degeneration score (which indicates the width of the severe cartilage thickness loss) compared to the OA-control rats suggested ongoing damage after withdrawal of the dietary supplement. In sum, all three modalities of oral SheaFlex75 treatment (SNO 1X, Late-SNO and SNO-stop) showed a trend to decrease of cartilage deterioration and had at least one significant reduction of the OARSI parameters as compared to age-matched OA-control.

**Table 1:** Histological scoring of OA knee joint.

	12^th^ week	24^th^ week			
	OA-Control (n = 4)	OA-Control (n = 5)	OA-SNO 1X (n = 4)	OA-Late SNO(n = 4)	OA-SNO-stop(n = 4)
Cartilage matrix loss 0% (mm)	1.755±0.191	1.953±0.132	*1.615±0.138	2.251±0.147	1.826±0.193
Cartilage matrix loss 50% (mm)	0.652±0.242	0.665±0.216	0.763±0.180	0.862±0.257	0.473±0.237
Cartilage matrix loss 100% (mm)	0.210±0.124	0.360±0.136	0.108±0.072	0.421±0.276	0.299±0.196
Medial Tibia Cartilage Degeneration Score	*5.25±1.146	8.10±0.795	6.75±0.726	6.5±1.134	7.125±0.549
Total cartilage degeneration width (mm)	*1.886±0.204	2.296±0.103	2.019±0.159	*1.347±0.169	*1.986±0.103
Significant cartilage degeneration width (mm)	0.680±0.257	1.123±0.123	*0.734±0.184	*0.668±0.225	0.774±0.212
Zonal depth ratio of lesions	0.311±0.051	0.421±0.055	0.399±0.042	0.388±0.061	0.412±0.038

Asterisk denote the statistical examination in comparison with 24^th^ week OA-Control using Student's t-test.

**Fig 4:**
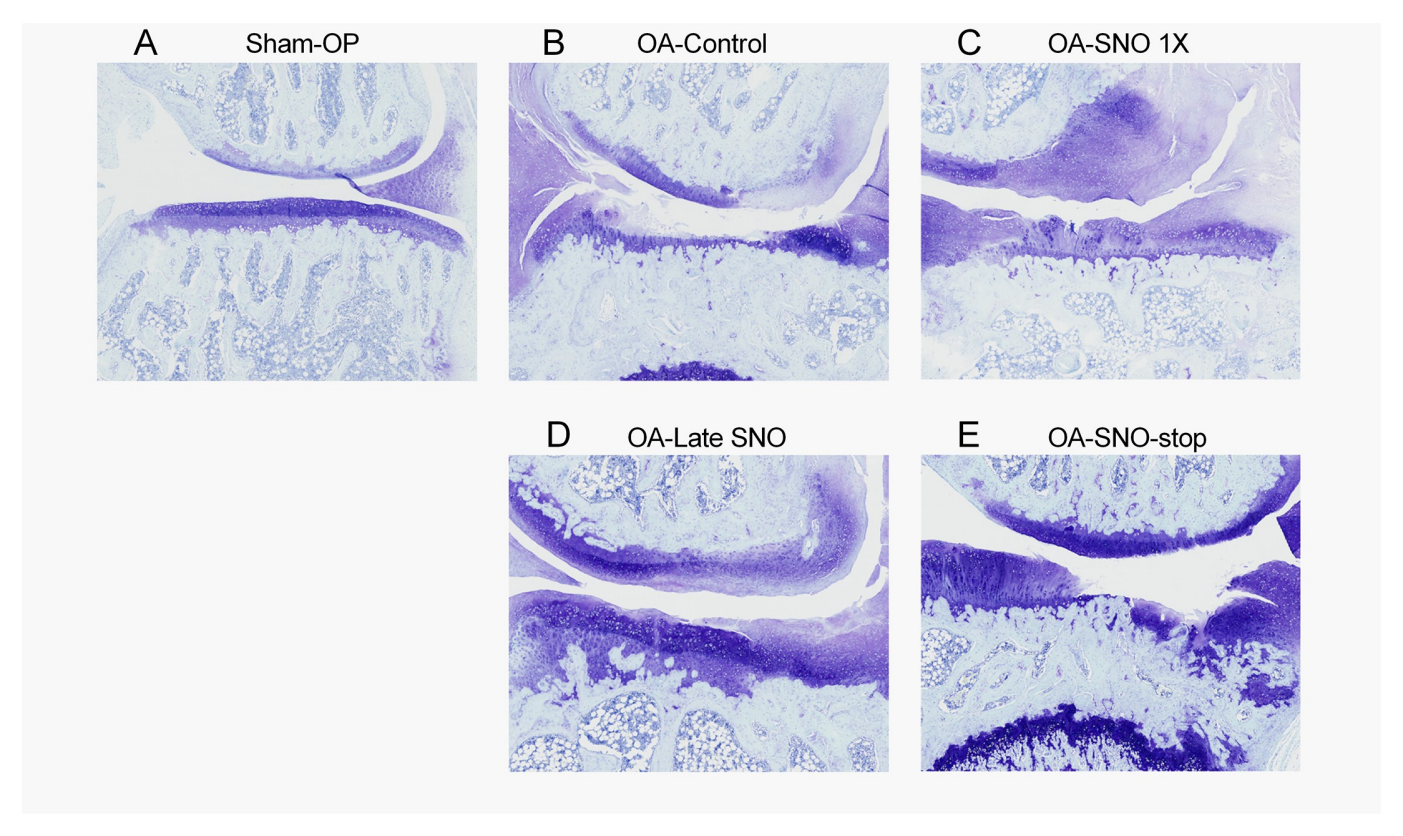
SheaFlex75 delays histopathological changes in the OA knee joint. Histological sections from OA knees from the sham-OP (A), OA-control (B), OA-SNO 1X (C), OA-Late SNO (D), and OA-SNO-stop (E) rats at week 24 post-surgery. The histological sections were processed with toluidine/fast green staining.

Fig 4 shows representative histological sections stained with toluidine/fast green. In the sham-OP group, both femoral and tibial cartilage remained intact after 24 weeks post-OP (Fig 4A). In contrast, the untreated OA-control rats presented loss of matrix and cellularity in the mild zone of the cartilage, resulting in exposure of the tidemark at the medial plateau of the tibia (Fig 4B). The OA-SNO 1X (Fig 4C) and OA-Late SNO (Fig 4D)-treated rats showed relatively conserved cartilage coverage in compared to OA-control, and less matrix loss with the tidemark unexposed. However, in the OA-SNO stop rats, some of the tidemark area was exposed, and the matrix and chondrocytes were compromised in the mild zone (Fig 4E).

### Long-term safety of oral SheaFlex75 administration in OA rats

**Fig 5:**
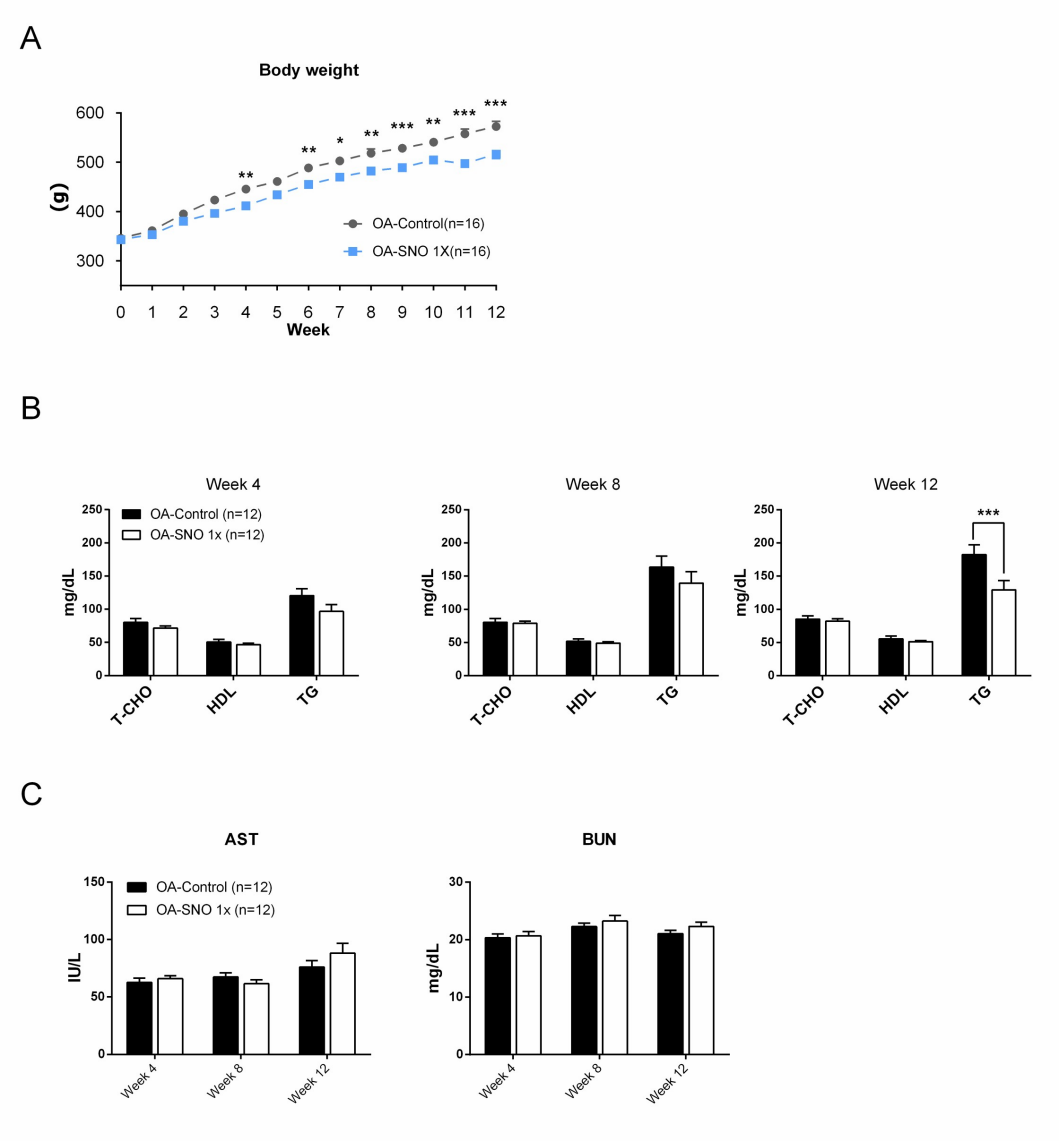
Time-course changes in the serum chemistry, lipid profile and body weight. (A) Body weight changes from 0–12 weeks in the OA-control and OA-SNO 1X rats. (B) Total cholesterol (T-CHO), HDL and triglyceride (TG) levels at weeks 4, 8 and 12 after ACLT+MMx surgery. (C) Time-course analysis of the serum AST and BUN levels in both the OA-control and OA-SNO 1X groups. Values are expressed as the mean±S.E.M. and two-way ANOVA and Sidaks’s multiple comparisons test were used to analyze the data. Asterisks denote significant differences compared to the OA-control rats: *P<0.05, **P<0.01, ***P<0.001.

Next, we evaluated the safety of long-term SheaFlex75 use in OA rats. Overall, the OA rats showed a static and gradual increase in body weight as they aged during the course of the experiment. Surprisingly, dietary ingestion of the SheaFlex75 1X dose produced a reduction in body weight gain (from 343.3±2.7 to 516.4±8.9 g) compared to the OA-control rats (from 345.9±4.2 to 572.3±10.7) (Fig 5A). Despite the oil property of this natural product, we found no change in total cholesterol and HDL, but the TG level was significantly reduced at week 12 in the OA rats treated with SheaFlex75 (182.42±14.7 vs 129.33±14.055 mg/dL) (Fig 5B). Both the AST and BUN levels remained within the normal range in both groups during the experiment (Fig 5C), suggesting the safety of the oral SheaFlex75 supplement under long-term dietary ingestion.

## Discussion

In our previous report [23], we found that oral supplementation of SheaFlex75 immediately after ACLT+MMx surgery showed a preventive effect for cartilage deterioration in ACLT+MMx rats. This early initiation of treatment showed a significant effect on attenuation of OA progression. However, previous reports claimed that ACLT+MMx-induced knee injury and focal cartilage changes were observed from the first week and that significant cartilage defects took 4–6 weeks to develop [27]. To better reflect the real conditions of human, people usually seek medical help after symptomatic OA developed, in our current study, we administered SheaFlex75 two weeks after ACLT+MMx, followed by a longer duration (24 weeks) of observation to mimic the real clinical condition of chronic OA progression.

In sum, our approach of starting treatment from day 14 or week 12 post-ACLT+MMx surgery allowed us to assess whether this natural product provides beneficial protection against symptomatic and histologically defined OA of the knee. In this study, we demonstrated a protective effect of SheaFlex75 in both the early and chronic stages of OA in rats. Reductions of joint swelling, hind paw pain and tibial cartilage degeneration were observed in the animals treated with shea nut oil. Continued use of this oil extract for up to 22 weeks produced a sustainable reduction in pain and cartilage degeneration, whereas later use or discontinuation of the product still provided protection, albeit to a lesser extent. The evidence in our present study together with that of our previous report [23] suggests that SheaFlex75, with its high triterpene concentration, is effective in delaying OA onset and progression in chronic OA rats. Additionally, earlier ingestion provides better cartilage protection and inhibition of OA progression.

Due to the complex triterpene concentrates (α-amyrin, β-amyrin, butyrospermol, lupeol and other triterpene alcohols) found in this product, we are unable to conduct a mechanistic study with a specific active compound in our animal model. Nonetheless, the pharmacological effects of the most abundant triterpenes (α-amyrin and β-amyrin) have been extensively studied and reviewed [28]. Otuki and colleagues demonstrated that a mixture of the triterpenes α-amyrin and β-amyrin produced consistent antinociception in inflammatory pain models [29]. Moreover, Kathryn and colleagues found that oral treatment with α-amyrin and β-amyrin (30 mg/kg) significantly attenuated mechanical and thermal hyperalgesia in the inflammatory/neuropathic pain models induced by complete Freund’s adjuvant/partial sciatic nerve ligation, respectively, via a mechanism based on the anti-inflammatory effects and activation of the CB1 and CB2 cannabinoid receptors [30]. A similar anti-inflammatory effect was demonstrated by Holanda and colleagues, who showed that the triterpenes α-amyrin and β-amyrin were effective in reducing inflammation in an acute periodontitis rat model [19]. Furthermore, the triterpenoids lupeol and α-amyrin were also reported to have inhibitory effects on serine protease (trypsin and chymotrypsin) activity, which decreased inflammation [31]. Increased collagenase activity and consequent collagen cleavage were demonstrated in human and animal studies of OA and were shown to be involved in disease pathogenesis and progression [32–35]. Consistent with our present findings, the high concentrations of α-amyrin, β-amyrin, lupeol and other triterpenes in this shea nut oil extract may be responsible for the reduction in OA inflammatory pain and may explain the attenuation of cartilage matrix loss and degeneration of structural integrity.

In addition, TGF-beta signaling and matrix metalloproteinases in the OA joints play an important role in the synthesis and degradation of extracellular matrix, respectively [36, 37]. Also, they were showed to have age-dependent activity and a temporal regulation during OA progression [38, 39]. Their expression pattern and activity in OA animal under SheaFlex75 supplementation remained to be explored. A dose/time-dependent study with more emphasis on the molecular analysis of the tissue specimens is mandatory for exploration of the potential anti-inflammatory mechanism of shea nut oil extract on inhibition of OA progression.

Chronic OA in the elderly is commonly accompanied by metabolic disorders, including obesity, diabetes, dyslipidemia and/or hypertension [40]. An emphasis on resolving metabolic disorders, such as weight reduction, could ameliorate the progression of OA [41–43]. Being overweight is an important risk factor for symptomatic OA progression, and weight reduction is a highly recommended strategy for OA management. In our experiment, the SheaFlex75 supplement ameliorated not only OA progression and cartilage loss but also body weight gain. Furthermore, we found that the rats provided with long-term supplementation with the shea nut oil extract SheaFlex75 for OA treatment showed no alterations in their blood AST, BUN, and cholesterol profiles compared to the OA controls. Moreover, a mild reduction in the serum TG level under the shea nut oil diet was previously reported, in which naïve hamsters fed the shea nut oil diet exhibited no alteration of plasma cholesterol, although a trend of reduction in the plasma triacylglycerol level was observed [44]. Similarly, in our OA rats, we found no alteration of cholesterol under long-term SheaFlex75 supplementation, but a significant reduction of the TG level compared to the OA controls was observed.

Notably, the reductions in both the TG level and body weight could be indirect effects of the oral supplement. OA-induced muscle wasting is observed in both humans and rats with OA due to avoidance of physical activity under chronic OA pain [45–47]. In contrast, a proper exercise intervention not only reduced cartilage degeneration in a rat OA model but also improved physical function in a human study [48, 49]. Active physical activity is as important as weight reduction for the prevention of OA knee deterioration. In our experiments, the reduction of OA pain induced by the high triterpene concentrates might have improved muscle strength and restored physical activity, leading to improvement in the overall metabolic state observed in our SheaFlex75-treated OA rats. Indeed, a previous human trial showed that SheaFlex75 relieved pain in OA patients and improved the patients’ muscle functions [21]; these observations further support our hypothesis. Future dynamic observations of weight bearing, daily activity, and food consumption could clarify this hypothesis and elucidate the cause-effect relationship of the observed phenotype.

A previous knee injury (e.g., an anterior cruciate ligament rupture) is highly correlated with the early onset of OA and its rapid progression [50, 51]. In our current study, we treated rats with SheaFlex75 on day 14 or week 12 post-ACLT+MMx surgery, which might mimic untreated OA with underlying structural damage of the joint. SheaFlex75 was shown to attenuate OA deterioration even in rats presenting with OA symptomatology and active histological damage. Surprisingly, cartilage protection was still observed after discontinuation of the oral supplement for up to 12 weeks, albeit to a lesser extent than that observed for the full regimen group. Nonetheless, the overall effect observed in the present study was less striking than that in our previous report [23], which showed significant cartilage scoring at week 12 post-OA induction in compared to our present design (S1 Table). One possible reason for this discrepancy was that our previous work, initiated treatment as early as immediately after surgery when the tibial cartilage had not yet been damaged; moreover, early inflammation of the knee joint could also be attenuated by the anti-inflammatory effect of the supplement. Based on these results and those of the current study, we conclude the importance of early management of OA as a preventive measure before the occurrence of histologically defined degeneration.

Based on the present study, preventive management is advised to delay/prevent OA onset and progression in patients; therefore, we suggest that SheaFlex75 may be an effective management strategy for symptomatic relief and cartilage protection in both acute and chronic OA patients.

## Supporting information

S1 Table Histological scoring of OA knee joint at 12th week post-surgery. (DOCX)
