# Supplementary material for: Oral shea nut oil triterpene concentrate supplement ameliorates pain and histological assessment of articular cartilage deterioration in an ACLT injured rat knee osteoarthritis model
Source: PLoS One. 2019 Apr 19;14(4):e0215812. doi: 10.1371/journal.pone.0215812 (PMC6474620; doi:10.1371/journal.pone.0215812)
Supplement: S1 Table — (DOCX) [file pone.0215812.s001.docx]

**S1 Table. Histological scoring of OA knee joint at 12^th^ week post-surgery.**

|  |  | | |
| --- | --- | --- | --- |
| 12^th^ week post-surgery | OA-Control (n=4) |  | OA-SheaFlex 75^TM^ (n=4) |
| Cartilage matrix loss 0% (mm) | 1.755±0.191 |  | 1.548±0.300 |
| Cartilage matrix loss 50% (mm) | 0.652±0.242 |  | 0.391±0.197 |
| Cartilage matrix loss 100% (mm) | 0.210±0.124 |  | 0.106±0.070 |
| Medial Tibia Cartilage Degeneration Score | 5.25±1.146 |  | 4.75±1.048 |
| Total cartilage degeneration width (mm) | 1.886±0.204 |  | 1.740±0.228 |
| Significant cartilage degeneration width (mm) | 0.680±0.257 |  | 0.425±0.236 |
| Zonal depth ratio of lesions | 0.311±0.051 |  | 0.268±0.055 |
